# Plasticity of Astrocytic Coverage and Glutamate Transporter Expression in Adult Mouse Cortex

**DOI:** 10.1371/journal.pbio.0040343

**Published:** 2006-10-17

**Authors:** Christel Genoud, Charles Quairiaux, Pascal Steiner, Harald Hirling, Egbert Welker, Graham W Knott

**Affiliations:** 1 Départment de Biologie Cellulaire et de Morphologie, Lausanne, Switzerland; 2 Department of Neurobiology, Harvard Medical School, Boston, Massachusetts, United States of America; 3 Faculté des Sciences de la Vie, Ecole Polytechnique Federale de Lausanne, Lausanne, Switzerland; Salk Institute for Biological Studies, United States of America

## Abstract

Astrocytes play a major role in the removal of glutamate from the extracellular compartment. This clearance limits the glutamate receptor activation and affects the synaptic response. This function of the astrocyte is dependent on its positioning around the synapse, as well as on the level of expression of its high-affinity glutamate transporters, GLT1 and GLAST. Using Western blot analysis and serial section electron microscopy, we studied how a change in sensory activity affected these parameters in the adult cortex. Using mice, we found that 24 h of whisker stimulation elicited a 2-fold increase in the expression of GLT1 and GLAST in the corresponding cortical column of the barrel cortex. This returns to basal levels 4 d after the stimulation was stopped, whereas the expression of the neuronal glutamate transporter EAAC1 remained unaltered throughout. Ultrastructural analysis from the same region showed that sensory stimulation also causes a significant increase in the astrocytic envelopment of excitatory synapses on dendritic spines. We conclude that a period of modified neuronal activity and synaptic release of glutamate leads to an increased astrocytic coverage of the bouton–spine interface and an increase in glutamate transporter expression in astrocytic processes.

## Abstract 2

After increased sensory activity in the somatosensory cortex, astrocytic processes expand to increase their coverage of the bouton-spine interface, and expression of astrocytic glutamate transporters is up-regulated as well.

## Introduction

Glutamate released into the synaptic cleft is cleared via a range of high-affinity transporters found on neuronal and astrocytic membranes. This clearance is important for limiting the effect that raised levels of glutamate may have in the extracellular space, including altered synaptic transmission and excitotoxic damage [1–5]. Astrocytes make a significant contribution to this uptake [6–8] via their glutamate transporters GLT1 (glutamate transporter 1, EAAT2) and GLAST (glutamate aspartate transporter, EAAT1), and this would appear to be dependent on the proximity of this glial cell's membranes to the synapse [9,10]. Astrocytic processes are highly dynamic [11], provide a physical barrier restricting the diffusion within the extracellular space [12], and reduce spillover of glutamate that may cause extrasynaptic transmission [10,13]. Little is known, however, about how changes in neuronal activity in the cortex could affect the astrocytic coverage of synapses and the expression of their glutamate transporters. In vitro, neuronal activity has been shown to be correlated with increased expression of the glial transporters [14], similarly in vivo, during development after long-term induction of epileptic seizures [15], as well as in the adult, after traumatic injury [16].

In the present study, we investigated how a peripheral stimulus that increases sensory activity affects the expression of the high-affinity glutamate transporters in the cortex, as well as the morphology of the astrocytic membranes that surround the glutamatergic synapses. We used a single whisker stimulation paradigm [17] in adult mice that alters cortical activity and induces significant physiological and morphological alterations in the corresponding region of the somatosensory cortex [18–20]. Using Western blot analysis on single barrel columns corresponding to the stimulated whisker, we found that expression levels of GLT1 and GLAST were significantly increased. The other predominant glutamate transporter in the neocortex, EAAC1 (excitatory amino acid carrier 1), found on neurons, showed no significant change. Serial section electron microscopy (EM) revealed a morphological plasticity of the astrocytic processes around excitatory synapses. This showed that altered sensory experience increased the envelopment of the bouton–dendritic spine interface, increasing their exposure to the site of glutamate release.

## Materials and Methods

### Whisker Stimulation Protocol

Mice were anesthetized with sodium pentobarbital (60 mg/kg, intraperitoneally), and a small ferrous metal piece (1.5-mm long, 0.2 mm in diameter) was then glued onto the left, C2 whisker, 3 mm from the skin surface. All other whiskers were left untouched. After recovery from anesthesia, they were placed for 24 h in the Lausanne whisker stimulator. This apparatus consists of a cylindrical cage 12.6 cm in diameter, surrounded by an electromagnetic coil that delivers magnetic field bursts at 9 Hz (see [17]). The stimulus intensity was chosen so that it did not cause the stimulated whisker to touch any of the whiskers surrounding it. Throughout the period of stimulation, the mice could move freely, with food and water available ad libitum. All procedures were reviewed and approved by the Office Vétérinaire Cantonal (Lausanne), in accordance with Swiss Federal Laws.

### Tissue Collection for GLAST and GLT1 Quantification

Forty-two adult female mice (6–8 wk of age, body weight, 25–35 g) from the ICR-derived NOR strain [21] were used for the quantitative protein expression analysis. A total of 27 mice received continuous whisker stimulation. Twelve of these mice were put back in their home cage for 4 d before tissue collection (4 d post stim group); the 15 remaining were processed immediately after the stimulation (stim group). Fifteen unstimulated mice were used as controls (unstim group).

**Figure 1:**
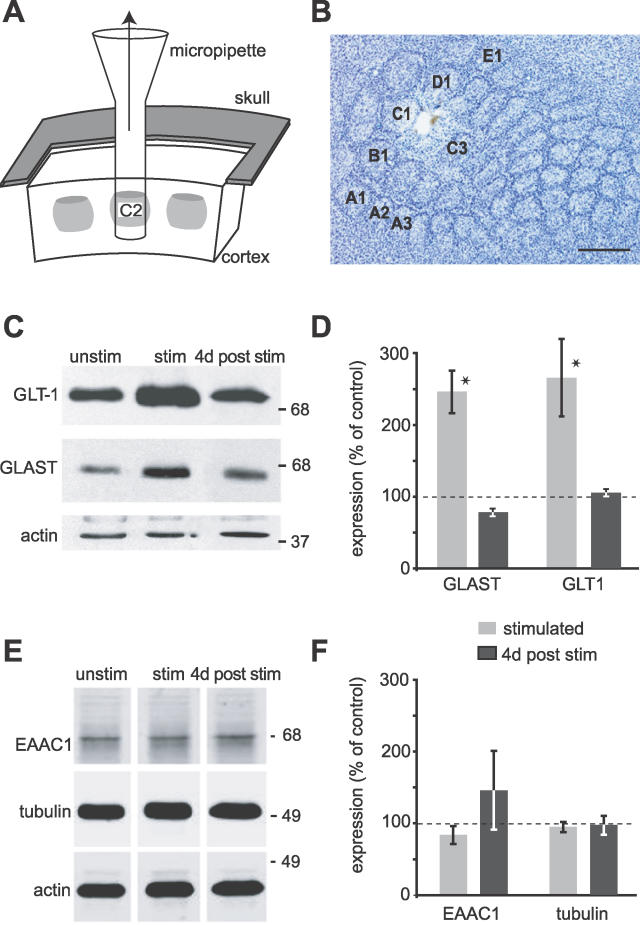
Up-Regulation of GLAST and GLT1 Protein Levels after Whisker Stimulation (A) A single barrel column (C2) was removed by aspiration through a glass micropipette, under sodium pentobarbital anesthesia. (B) Tangential section of the barrel cortex, Nissl stained, shows the location of the excised barrel column. A clear hole can be seen in the section in the region of barrel C2, with the neighboring barrels intact. (C) Representative immunoblot microassay of C2 columns dissected immediately after 24 h of C2 whisker stimulation (stim), 4 d after stimulation (4 d post stim), and from unstimulated mice (unstim). Blot was probed for GLAST, GLT1, and actin, and indicates an increase in GLAST and GLT1 levels after 24 h of whisker stimulation, but not 4 d later. (D) These changes were quantified using densitometry with the values being normalized against the actin levels. Results were expressed as percentages of levels in unstimulated mice (100%) and statistically analyzed with a Tukey studentized range test, *p* < 0.01; error bars indicate SD. Scale bar in (B) indicates 0.5 mm. (E) Representative immunoblots from animals treated as in (C), and analyzed for protein levels of EAAC1, tubulin, and actin. (F) Quantification of the immunoblot signals revealed no significant alteration in EAAC1 levels in stimulated animals. EAAC1 and tubulin values were normalized on actin levels, and expressed as % of level in control animals. Note that the relative level of tubulin was unchanged by the stimulation. Error bars indicate SD.

Tissue removal took place under sodium pentobarbital anesthesia (60 mg/kg, intraperitoneally). The animal was placed in a stereotaxic frame that provided a continuous flow of oxygen in front of the nose. Body temperature was maintained at 37 °C by a rectal thermistor-controlled heating pad. After skin incision, a craniotomy of the right parietal bone exposed the cortex above the posteromedial barrel subfield region. The cortical representation of the C2 whisker was mapped using multiunit recordings with carbon microelectrodes and manual deflection of the contralateral whiskers. Units in layer IV of a given cortical barrel respond faster and stronger to one particular whisker; the so-called principal whisker [22]. Once the C2 barrel column was located, a glass micropipette with internal diameter of 200 μm, corresponding to the size of the C2 barrel, was then lowered perpendicular to the pial surface, with a calibrated three-dimensional (3D) microdrive, to a depth of 800–1,000 μm. Tissue in the pipette was then expelled with the use of a pipette bulb and gentle pressure (Figure 1A). The tissue was then washed in PBS (phosphate buffer saline; 0.01 M), transferred to buffer A (see below), and cooled immediately to −80 °C. After barrel column removal, animals were transcardially perfused with 10% formalin in 0.9% NaCl. The brain was dissected from the skull and postfixed in the same fixative overnight, cryoprotected (30% sucrose, in 0.1 M PB [phosphate buffer]), and 40-μm sections were cut from the right hemisphere, tangential to the barrel cortex, with a freezing microtome. These sections were then Nissl-stained with cresyl violet to confirm the exact location of the dissected tissue (Figure 1B).

### Western Blots and Quantification Method

Single cortical columns that had been removed from individual mice were pooled together to make a total of four samples in each of the three groups: unstim, stim, and 4 d post stim. In the unstim and 24 h stim groups, each sample consisted of three columns pooled together. In the 4 d post stim group, samples consisted of two or three columns. These samples were re-suspended and immediately homogenized together in buffer A (0.32 M sucrose, 10 mM HEPES/KOH [pH 7.4], 10 mM DTT) that contained the protease inhibitors PMSF, aprotinin, leupeptin, and pepstatin. Denaturation was made by boiling for 10 min in an equal volume of buffer B (2% SDS, 60 mM Tris [pH 6.8], 10% glycerol, 5% 2-mercaptoethanol). A total of four independent Western blots were made of each of the four samples from the three groups. Samples were electrophoretically separated by SDS PAGE (sodium dodecyl sulfate polyacrilamide gel). Proteins were then transferred to Protran BA 83 nitrocellulose membranes, which were then incubated for 1 h with blocking buffer, and probed at 4 °C overnight with antibodies against the following proteins: monoclonal:tubulin (1:4,000; Sigma, St. Louis, Missouri, United States), actin (1:2,000; Roche, Basel, Switzerland), polyclonal: GLT1 (0.05 μg/ml, [23]), GLAST (0.05 mg/ml, [24]), EAAC1 (1:1,000; Alpha Diagnostics, San Antonio, Texas, United States). GLT1 and actin, or EAAC1 and actin (Figure 1) were first detected, followed by stripping of the membrane and reprobing for GLAST or tubulin, respectively. We used peroxidase-conjugated secondary antibodies followed by detection of the chemoluminescent bands (RPN 2106; Amersham, Little Chalfont, United Kingdom) by Kodak X-ray films (Rochester, New York, United States) and semi-quantitative evaluation by densitometrical analysis using the National Institutes of Health NIH Image software. We also used IRDye-coupled secondary antibodies (Rockland, Gilbertsville, Pennsylvania, United States) followed by quantitative detection of the fluorescent bands using the Odysee system (Li-Cor, Lincoln, Nebraska, United States). Actin and tubulin are the two “housekeeping” proteins that we used to analyze modifications in the expressions of the three glutamate transporters after stimulation. Compared to tubulin levels, actin levels remained stable in control and stimulated animals (unstim, 101.4 ± 7.5%; 24 h stim, 108 ± 10.8%; and 4 d post stim, 104.5 ± 11.9%) [24]. GLT1, GLAST, and EAAC1 levels were normalized to actin.

### Serial Section EM

The preparation of the material used to make a morphometric analysis of spines and adjacent structures was carried out as described previously [20], and is briefly outlined below. A total of 12 female NOR mice were used at 6 wk of age. The right C2 whisker of six of these animals was stimulated for a period of 24 h (see above), and the remaining six were used as the unstimulated animals (unstim group).

### Fixation, Embedding, and Image Acquisition

Immediately after removal from the stimulator, mice were anesthetized (as above) and perfused with 300 ml of fixative (2.5% glutaraldehyde and 2% paraformaldehyde in cacodylate buffer, 0.1M [pH 7.4]). One hour after perfusion, the brain was removed and left in the same fixative for 1 h before 60-μm vibratome sections were cut (Leica VT100; Wetzlar, Germany) tangentially from the barrel cortex. These sections were then washed in cacodylate buffer, postfixed in 1% osmium tetroxide in 0.1 M cacodylate buffer, dehydrated in alcohol, and embedded between silicon-coated glass slides in Durcupan resin (Fluka, Buchs, Switzerland). Once the resin had cured, the C2 barrels could be identified and a trapezoid block prepared encompassing this region. Series of between 60 and 100 silver/grey (60-nm thickness) thin sections were cut on single-slot grids bearing a Formvar support film that had been lightly carbon coated.

Serial images of the neuropil were collected using a digital camera (MegaView III, SIS, Munich, Germany) in a Philips CM12 electron microscope using a filament voltage of 80 kV, aligned using Photoshop software (Adobe, San Jose, California, United States), and analyzed in the Neurolucida software (Microbrightfield, Williston, Vermont, United States).

### The 3D Analysis of Dendritic Spines and Astrocytic Membranes

In a previous study, we have shown that this whisker stimulation paradigm causes an increase in the density of asymmetric synapses on spines and also an increase in the density of inhibitory synapses on spines [20]. Similar analysis was performed on the volumes of neuropil analyzed in the current study. This analysis found equivalent modifications in synaptic densities as the earlier study. We subsequently reconstructed all the spines, together with the surrounding astrocytic processes in 3D using the Neurolucida software. Only those that were completely within the sample volume were included. A total of 403 spines were reconstructed from six unstimulated mice in a total of 826 μm^3^ of neuropil, and 405 spines from six stimulated mice in a total of 674 μm^3^ of neuropil.

**Figure 2:**
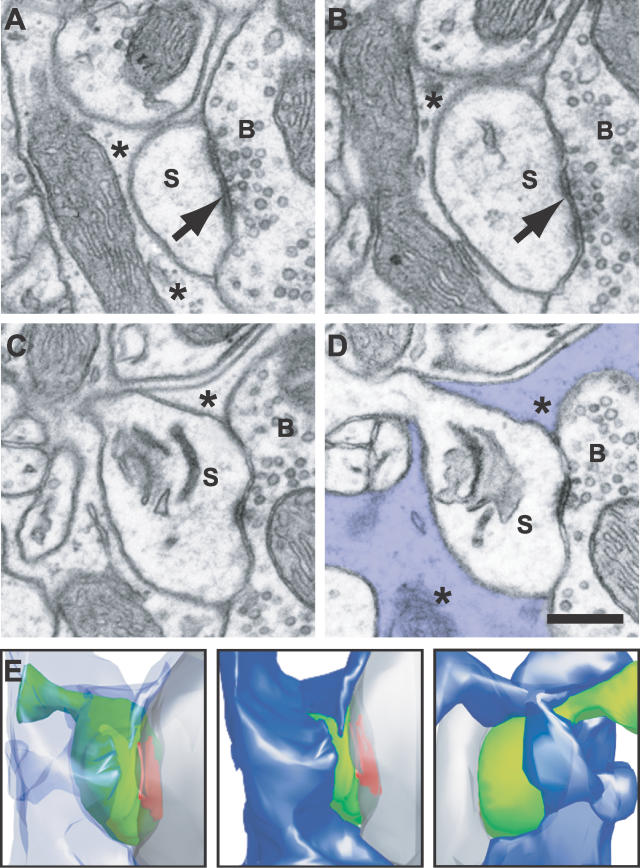
Serial EM Images, and Reconstruction, of a Dendritic Spine from a C2 Barrel Hollow (A–D) Show four micrographs from a series of 18 that were used to reconstruct the entire dendritic spine (S); making an asymmetric synaptic contact with a bouton (B) (arrow in [A] and [B]). The astrocytic element that surrounds this spine is marked with an asterisk (*) and can be seen to be closely associated with the interface between the spine head and the axonal bouton. Scale bar in (D) indicates 0.5 μm. (E) Shows the corresponding 3D reconstruction of this spine (green), bouton (grey), PSD (red), and astrocyte (blue) in three images below. The left-hand image shows the spine in the same orientation as the above micrographs, with a transparent astrocyte revealing the shape of the spine beneath; the middle image is in the same orientation, but the astrocyte is now opaque, showing the degree to which the spine is covered. The right-hand image shows the spine and covering astrocyte, viewed after a 180° rotation around the *y* axis.

The following spine parameters were measured: spine volume, spine surface area, spine surface area apposed by astrocytic membrane, and area of the postsynaptic density (PSD; Table 1). The size of the astrocytic envelopment of the bouton–spine interface was quantified by measuring the total perimeter of the bouton face that contacted the spine, and the proportion that was occupied by the astrocyte. This is illustrated in Figure 2 with micrographs through a single spine (Figure 2A–2D), and with a 3D reconstruction of this region (Figure 2E) showing the surrounding astrocyte and the bouton–spine interface.

**Table 1:**
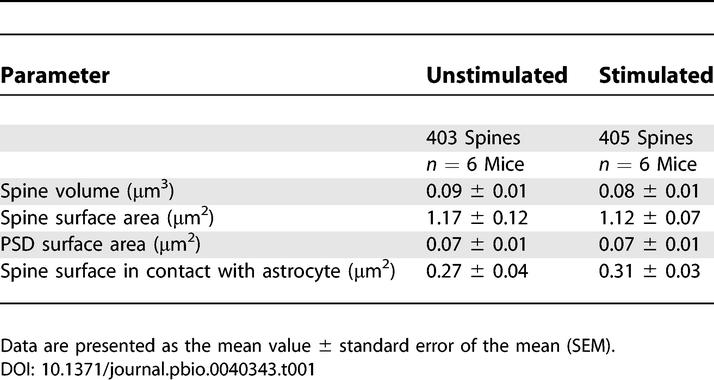
Morphometric Data of Reconstructed Spines

### Statistical Analysis

**Figure 3:**
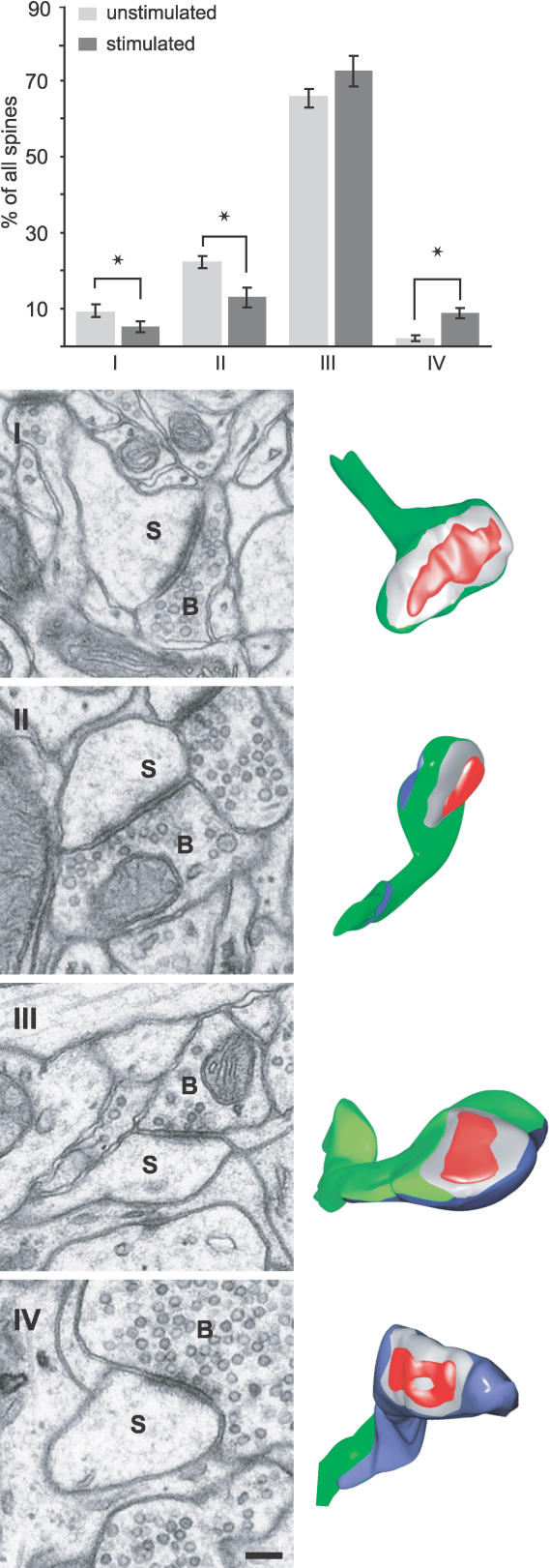
Sensory Stimulation Increases Percentage of Spines Whose Bouton–Spine Interface Is Surrounded by Astrocyte The histogram shows the distribution of four classes of spines, sorted according to their degree of contact with the astrocyte (see examples of classes I–IV), expressed as mean ± standard error of the mean (SEM) (unstimulated, *n* = 6 mice; stimulated. *n* = 6 mice). The percentage of spines in class IV, whose bouton–spine interface is completely surrounded by an astrocytic element, was increased significantly in stimulated mice (*p* < 0.03). Dendritic spines were classified into four classes, I–IV, based on the arrangement of the astrocyte at their surface. Electron micrographs of spines of each class are shown, as well as the 3D reconstruction of the whole spine to the right. (spines are indicated with an S and axonal boutons, B). Examples of spines in classes I–IV, and their reconstructions, are shown. Scale bar in lower micrograph represents 200 nm.

Statistical analysis has been performed with the SAS statistical package (SAS Institute, Cary, North Carolina, United States). The distribution of the morphometric parameters were tested for normality (Univariate procedure, SAS Users Guide, Base, 1989) and the means between groups were compared with a multivariate analysis of variance (GLM procedure, SAS/STAT User's Guide, version 6, 1989). The distribution frequency for the four classes of spines (Figure 3) was compared between the two groups (unstimulated and stimulated) using a chi-square (χ^2^) test.

## Results

### Modification of Protein Expression: Increased Expression of Astrocytic Glutamate Transporters GLAST and GLT1 after Whisker Stimulation

By lowering a micropipette into the neocortex and applying a slight negative pressure, we were able to extract an entire barrel column (Figure 1A), and collect samples from the three groups of mice. This method proved successful in removing the electrophysiologically identified barrel column, which was verified by fixing and histologically processing the brain (Figure 1B). This region was reconstructed from the tangential Nissl-stained serial sections through the entire cortical thickness (Figure 1B) using the Neurolucida software. Western blot analysis (Figure 1C and 1D) shows that after 24 h of whisker stimulation, GLAST and GLT1 expression increased 2.46-fold and 2.65-fold of the unstimulated value (GLAST: 246 ± 20.9% mean ± standard deviation [SD]; GLT1: 265 ± 38.1%; *p* < 0.01, Tukey honestly significant difference [HSD]). Four days after the stimulation had been stopped, both transporter levels returned to control values. When similarly treated animals were analyzed for the level of the neuronal glutamate transporter EAAC1, we did not detect a significant change in the stimulated group compared to unstimulated (82% ± 14%; *p* = 0.27) or 4 d after stimulation (145% ± 58%; *p* = 0.17).

These results prompted us to explore whether the localization of the transporters changed with stimulation. Using pre-embedding immunohistochemistry, we found that GLT1 and GLAST were always located in the astrocytic compartment (Figure S1), as shown in other studies [25,26]. There was also consistently strong staining in astrocytic profiles that were in close proximity to asymmetric synapses. In contrast, EAAC1 was found exclusively in neurons, mostly in the postsynaptic compartment, and sensory stimulation did not appear to alter this (Figure S1).

### Morphometric Analysis of Dendritic Spines and Astrocytic Contacts

From the serial EM images of neuropil in the barrel hollows, the total astrocytic volume was measured by delineating all the astrocytic processes in every image. This was carried out in the volumes of three unstimulated mice (total volume analyzed, 555.58 μm^3^) and three stimulated mice (413.41 μm^3^). These volumes did not include any somata of neurons, glia, or endothelial cells; only the axons, dendrites, and astrocytic processes. In the unstimulated mice, astrocytes occupied 8.49 ± 1.70% of the total volume, and 9.44 ± 3.08% of the total volume in stimulated animals. The total surface area of the astrocyte within these volumes was 121.36 ± 4.03 μm^2^/100 μm^3^ in the unstimulated, and 132.79 ± 15.25 μm^2^/100 μm^3^ in stimulated mice. Whisker stimulation, therefore, does not appear to alter the proportion of astrocyte within the affected neuropil in terms of either the total volume (*p* = 0.66) or total membrane surface area (*p* = 0.27).

This result characterizes the overall glial content of the neuropil, but does not provide information about possible modifications of the astrocytic processes around glutamatergic synapses. Because the majority of glutamatergic synapses are located on dendritic spines in the neocortex, we reconstructed all complete spines present within the volumes of neuropil from the 12 mice (*n* = 6 unstimulated mice, 403 spines; *n* = 6 stimulated mice, 405 spines) and determined the surface area that was covered by astrocytes.

This analysis (Table 1) showed that stimulation had no effect on the following parameters: spine volume, spine surface area, area of the PSD, and area of astrocytic membrane in contact with the dendritic spine (*p* > 0.5 for all parameters).

### Morphology of the Astrocytic Envelopment of the Bouton–Spine Interface

In view of the significant changes in the transporter expression on the astrocyte, we focused the morphological analysis on the region around the synapse; the interface between the spine and axonal bouton, and the amount of astrocyte that occupies this region. Figure 2 shows electron micrographs (Figure 2A–2D) through a dendritic spine and its 3D reconstruction (Figure 2E). In this reconstructed example, a large proportion of the spine surface is in direct contact with the astrocyte (colored in blue in Figure 2D and 2E), which also occupies a significant part of the interface between the bouton and spine. In general, spines in barrel hollows show a large diversity in the portion of their surface that contacts astrocytes; some showed almost complete astrocytic coverage, whereas others were completely devoid of contact. Therefore, we divided the reconstructed spines into four classes (illustrated in Figure 3): class I, spines without any contact with astrocytes; class II, spines with astrocytic contact that did not contact the bouton–spine interface; class III, spines in contact with astrocyte which also partially encircled the bouton–spine interface (as in the example shown in Figure 2); and class IV, spines in which the astrocytic coverage almost completely (>95%) surrounded the bouton–spine interface.

In both unstimulated and stimulated groups, more than 90% of all the spines in the sampled volumes were in contact with astrocytic processes to some degree (classes II, III, and IV; mean per animal: unstimulated, 90.5 ± 1.6%; stimulated, 94.9 ± 1.9%). A large majority of the spines had an astrocytic process at the bouton–spine interface (classes III and IV; average per animal: unstimulated, 68.1 ± 1.7%; stimulated, 81.7 ± 3.5%). A two-way frequency test revealed that the distribution of these classes was significantly different between the unstimulated and stimulated groups (*p* < 0.001, chi-square). There was a significant increase in the percentage of spines in which the bouton–spine interface was completely occupied by an astrocytic process (class IV: unstimulated, 1.98 ± 0.81%; stimulated 8.64 ± 1.28%, *p* = 0.03). As well as a significant decrease in the numbers of spines in classes I and II (*p* < 0.05). This result implies that stimulation encourages an astrocytic encroachment of the bouton–spine interface, reflected in the greater number of spines that have this region completely enveloped.

**Figure 4:**
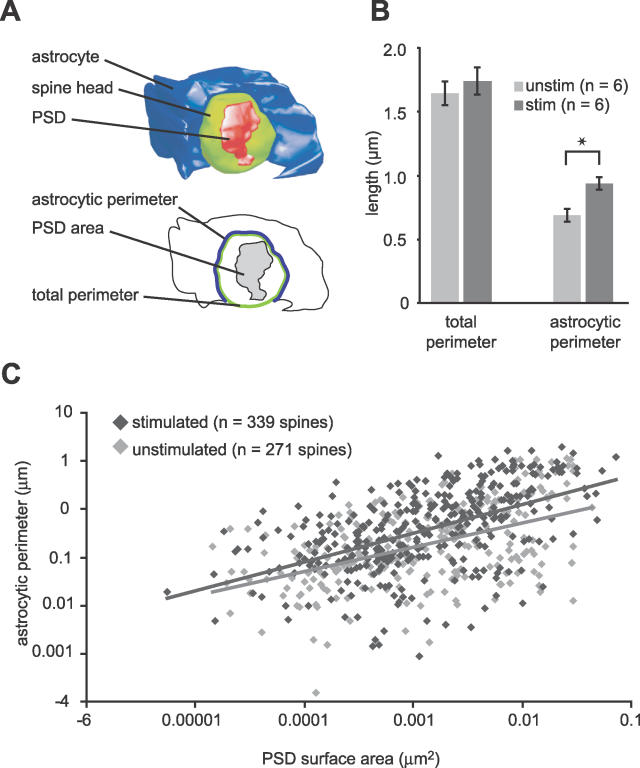
Whisker Stimulation Increases the Astrocytic Participation at the Bouton–Spine Interface (A) Shows a 3D reconstruction of a spine head (green), its PSD (red), and the associated astrocyte (blue). The orientation of the structure shows the region occupied by the axonal bouton (removed). The line drawing below shows the parameters measured: the total perimeter of the interface between the bouton and the spine, and the part of this perimeter that is occupied by the astrocyte, the astrocytic perimeter. (B) Stimulation did not change the degree of contact between bouton and spine, measured by the total perimeter (*p* > 0.5). However, the amount of the perimeter occupied by the astrocyte was significantly increased (*p* < 0.0001), using mean values per animal. (C) Correlation between the length of the perimeter that is occupied by astrocytic membrane and the PSD surface area on spines in unstimulated (light grey diamonds, *n* = 271; *p* < 0.001, *R^2^* = 0.68) and stimulated neuropil (dark grey diamonds, *n* = 340; *p* < 0.001, *R^2^* = 0.73).

We then asked whether stimulation affected only the astrocytic structural conformation around the glutamatergic synapses, or also the interaction between the spine and bouton. This coverage was analyzed by measuring the perimeter of the interface between the spine and bouton (Figure 4, total perimeter). We included all the spines analyzed from the twelve mice (classes I, II, III, and IV: unstimulated, 403 spines, *n* = 6; stimulated, 405 spines, *n* = 6). This varied from 0.49 μm for the smallest spines to 4.33 μm for the largest. Stimulation does not change the size of the perimeter, and therefore, the degree of contact between spine and bouton does not change (unstimulated, 1.65 ± 0.09 μm; stimulated, 1.74 ± 0.09 μm; *p* > 0.5). If we look at the effect of stimulation on the part of the perimeter occupied by the astrocyte, we find a significant increase (unstimulated 0.70 ± 0.12 μm; stimulated 0.94 ± 0.12 μm; *p* < 0.0001). For this analysis we used the spines in classes III and IV (unstimulated spines = 271, *n* = 6 mice; stimulated spines = 340, *n* = 6 mice; Figure 4B). Therefore, stimulation increased the amount of astrocyte apposed to the bouton–spine interface. This shows that stimulation causes the astrocytic processes to adopt a more intimate contact with the region through which glutamate transmission occurs: the bouton–spine interface, a parameter that is unaffected by the stimulation.

To allow for a physiological interpretation of these results, we then analyzed how this astrocytic envelopment was correlated with the size of the synapses on spines in classes III and IV. Linear regression analysis shows in both groups a correlation between the PSD size and astrocyte envelopment (Figure 4C, unstimulated *p* < 0.001, *R^2^* = 0.68; stimulated *p* < 0.001, *R^2^* = 0.73.

## Discussion

This study shows that an increase in sensory activity increases the expression of the astrocytic glutamate transporters GLT1 and GLAST in the corresponding region of the primary somatosensory cortex, without affecting the neuronal glutamate transporter EAAC1. There is also a morphological plasticity of the astrocytic membranes in the vicinity of the glutamatergic synapses, with an increase in their coverage of the bouton–spine interface.

The stimulation paradigm that we used, in which a single whisker moves passively and independently of the others, changes the firing pattern of layer IV neurons [20], increases GAD (glutamic acid decarboxylase) immunoreactivity [18], and increases the density of inhibitory synapses on dendritic spines [20]. This suggests a response by the cortex that balances the effects of chronic overstimulation to the layer IV circuitry by the incessant activation of the whiskers. We now show, in addition to an up-regulation of the inhibitory system in the cortex and an increase in the density of asymmetric synapses on spines, a plasticity of astrocytic processes close to asymmetric synapses, and the expression of their glutamate transporters; both factors implicated in the removal of glutamate released into the synaptic cleft.

These changes could reflect a plasticity that helps the cortex to guard against the effects of glutamate accumulation in the extracellular space. The termination of glutamate activity is brought about through its diffusion and uptake via its transporters, and its accumulation will not only affect the fidelity of signaling at one synapse, but may cause its “spillover” into neighbor synapses and extrasynaptic receptors [27,28]. This could then render the network susceptible to events such as epileptiform activity. The excessive receptor activation could also lead to excitotoxic damage [5,29].

Changes in neuronal activity and glutamate release have been shown to affect glutamate uptake [30,31]. In the mammalian hippocampus, the induction of long-term potentiation (LTP) in vitro, and fear conditioning in vivo, cause increased glutamate uptake [32] that is Na^+^ dependent. This is partly due to a translocation of the neuronal transporter EAAC1 from the cytosol to the membrane, occurring within an hour after stimulation [32]. Under these conditions GLT1 and GLAST did not appear to undergo a similar post-translational modification. On a longer time scale, however, the expression levels of GLT1 and GLAST have been shown, in vitro, to depend on the level of neuronal activity [14]. Here we now show, in vivo, that after 24 h of altered sensory stimulation, their expression increases, whereas the expression levels of the neuronal transporter EAAC1 remain unchanged.

This unaltered EAAC1 expression may be a reflection of its relative importance in glutamate clearance in the sensory cortex. Injections of antisense oligonucleotides against the GLT1 and GLAST into the lateral ventricles of rats causes neurodegeneration in the cerebral cortex, but similar blockage of EAAC1 synthesis had only mild neurotoxic effects [5]. This study, therefore, suggests that in the cerebral cortex, EAAC1 does not play a leading role in protecting against excess glutamate. In the neocortex, GLT1 and GLAST inhibition increases extracellular glutamate, which affects the network excitability [33]. Increase in epileptic activity has also been seen when only GLT1 expression is lowered [34,35]. Similar dominance of the glial transporters over EAAC1 was also shown in mutation studies [36]. All the evidence supports the idea that regulating the expression of GLT1 and GLAST during a period of increased glutamate release, e.g., during increased sensory stimulation, is necessary for avoiding any effects of glutamate build-up.

Studies exploring glutamate uptake and its influence on synaptic transmission, however, do not show such a major influence by the astrocyte in all brain regions. Astrocytic transporters are effective in altering mGluR1 occupancy on hippocampal interneurons, with EAAC1 having little effect [37]; as well as on parallel fiber–Purkinje cell synapses in the cerebellum [38]. However, between climbing fibers and Purkinje neurons, EAAC4 appears to be the main contributor [39]. And, on hippocampal CA1 pyramidal cells, EAAC1 slows the decay of NMDA currents, whereas GLT1 and GLAST having little effect [40].

Predicting the contribution of the different transporters in glutamate uptake is not simple, and other factors need to be considered. The positioning of transporters close to the site of glutamate release is paramount if they are to participate in uptake. In the hippocampus, where glial coverage is widely variable, 57% of excitatory synapses have astrocytic elements at their perimeter [13], and glial transporter blockade has a significant effect [37]. Here, in layer IV of the somatosensory cortex, we show that two thirds of the spines have at least some astrocytic membrane at the bouton–spine interface, and we could, therefore, speculate that the glial transporters play a significant role.

If the amount of astrocytic membrane encircling the synapse determines the degree of uptake, then larger synapses would perhaps have more astrocytic envelopment. We cannot measure glutamate released directly at the synaptic level, but previous work shows that the number of docked vesicles correlates with the number of vesicles that are ready for release [41], as well as with the size of the synapse [42]. Therefore, synapse size is likely to be a good indication of glutamate release per synaptic event. We show a correlation with the amount of astrocytic envelopment and the size of the PSD. This could simply be the consequence of a random distribution of the astrocyte, which would increase its envelopment for larger synapses. After stimulation, however, we find no change in the size of the bouton–spine interface, size of the PSD, or the total contribution made by the astrocyte to the neuropil that we sample, but a significant increase in astrocytic envelopment that remains proportional to synaptic size. Therefore, synapses in the stimulated regions would appear to have a greater ability to attract astrocytic membrane, a possible reflection of their greater activity, the consequences of which could be to maintain the fidelity of synaptic communication and avoid the effects of raised extracellular glutamate. This protective nature of astrocytes has also been shown after a central nervous system lesion in which their activation and hypertrophy limits the degree of synaptic loss [43]. Their altered morphology was shown with light microscopy by intracellular labeling, which also reveals their highly ramified structure and their non-overlapping domains [44,45]. This is an important consideration in the study presented here because it shows that, in each of our sampled volumes, we may be measuring the astrocytic processes belonging to as few as one single astrocyte that is capable of surrounding many synapses.

The current study shows that sensory stimulation affects both the morphology of astrocytic processes that lie on the immediate vicinity of glutamatergic synapses, as well as the expression of their glutamate transporters. So far we have discussed these results in the context of the astrocytes' ability to remove glutamate from the extracellular space. However, numerous experiments indicate that the role of these glial cells may be far greater than just as scavengers of this major neurotransmitter. Astrocytes receive and respond to direct signals from neurons (for review see [46]). They express both metabotropic glutamate receptors (mGluRs) and ionotropic glutamate receptors (iGluRs) [47,48], and neuronal stimulation provokes increases in their intracellular calcium [49]. Although much of this work was carried out in ex vivo slices, a recent study has demonstrated that whisker activation also evokes increases in astrocytic cytosolic calcium within the barrel cortex [50]. Moreover, this was shown to be caused by glutamate release, via spillover or ectopic release, and mediated by mGluRs. Glutamate activation of astrocytes can provoke the release of neurotransmitters and neuromodulators, including: glutamate [51], prostaglandin [52], and TNFα [53,54]. This, combined with the data showing how astrocytes are able to exert control over excitatory and inhibitory transmission [55,56], as well as over the dilation of the arterioles [57], suggests that a greater physical coupling between astrocytes and neurons, as shown here, would have important consequences for the way in which the cortex responds to alterations in sensory activity.

## Supporting Information

Figure S1 Localization of Glutamate Transporters in the Neuropil Pre-embedding immunocytochemistry in stimulated neuropil showed that GLT1 (upper left panel) and GLAST (upper right panel) immunoreactivity was confined to astrocytic processes (star), shown here surrounding a dendritic spine head (S) and axonal bouton (B). EAAC1 staining (three lower panels), however, was confined to the neuronal compartments, particularly dendritic spines (S) and dendritic shafts (DS), with the astrocytic compartments showing no labeling (star). Micrographs are the same magnification, and the scale bar indicates 0.5 μm.
(7.9 MB PDF)

## Abbreviations

3D: three dimensional
EM: electron microscopy
PSD: postsynaptic density
SD: standard deviation
