# Supplementary material for: Plasticity of Astrocytic Coverage and Glutamate Transporter Expression in Adult Mouse Cortex
Source: PLoS Biol. 2006 Oct 17;4(11):e343. doi: 10.1371/journal.pbio.0040343 (PMC1609127; doi:10.1371/journal.pbio.0040343)

Supplemental Figure 1

Localization of glutamate transporters in the neuropil.
Pre-embedding immunocytochemistry in stimulated neuropil showed that GLT1 (upper left panel) and GLAST (upper right panel) immunoreactivity was confined to astrocytic processes (star), shown here surrounding a dendritic spine head (**S**) and axonal bouton (**B**). EAAC1 staining (3 lower panels), however, was confined to the neuronal compartments, particularly dendritic spines (**S**) and dendritic shafts (**DS**), with the astrocytic compartments showing no labelling (star). Micrographs are the same magnification and scale bar is 0.5 µm.

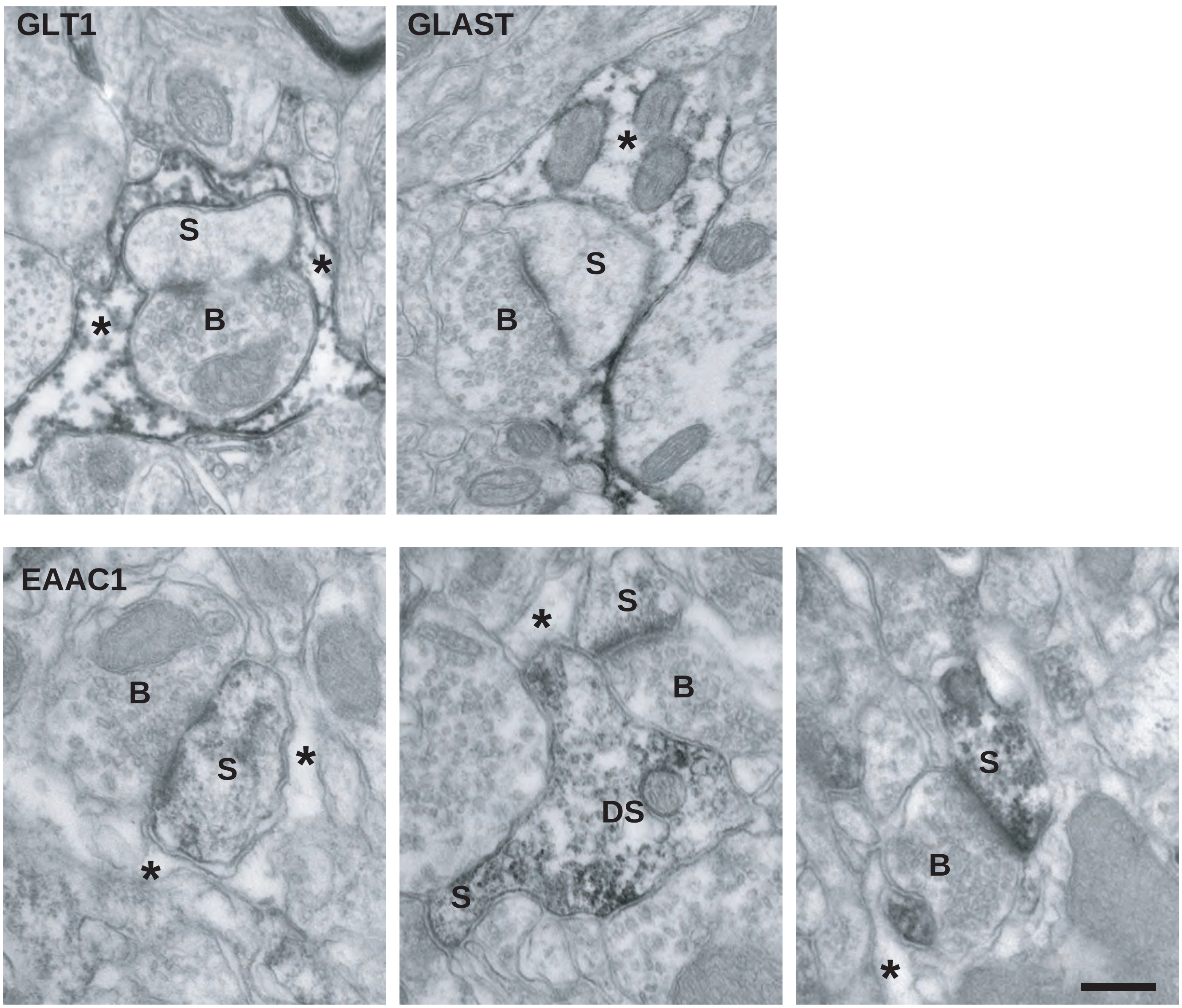

Supplement: Supplementary file 1 [file pbio.0040343.sg001.pdf]
